# Type 2 diabetes mellitus increases the severity of non-fatal injuries, but not the risk of fatal injuries, among driver victims of motor vehicle crashes in Taiwan

**DOI:** 10.4178/epih.e2022076

**Published:** 2022-09-16

**Authors:** I-Lin Hsu, Wen-Hsuan Hou, Ya-Hui Chang, Chung-Yi Li

**Affiliations:** 1 Department of Surgery, National Cheng Kung University Hospital, College of Medicine, National Cheng Kung University, Tainan, Taiwan; 2 College of Medicine, National Cheng Kung University, Tainan, Taiwan; 3 Department of Geriatrics and Gerontology, National Cheng Kung University Hospital, National Cheng Kung University, Tainan, Taiwan; 4 School of Gerontology and Long-Term Care, College of Nursing, Taipei Medical University, Taipei, Taiwan; 5 Department of Public Health, College of Medicine, National Cheng Kung University Tainan, Taiwan; 6 Department of Public Health, College of Public Health, China Medical University, Taichung, Taiwan; 7 Department of Healthcare Administration, College of Medical and Health Science, Asia University, Taichung, Taiwan

**Keywords:** Diabetes mellitus, Motor vehicles, Traffic accidents, Cohort studies, Injury severity, Mortality

## Abstract

**OBJECTIVES:**

Limited information is available on whether diabetes increases the severity of injuries from motor vehicle crashes (MVCs). This study aimed to investigate the association of type 2 diabetes with injury severity among driver victims of MVCs.

**METHODS:**

This cohort study involved 75,737 adult driver victims with type 2 diabetes from Taiwan’s Police-Reported Traffic Accident Registry in 2015–2017, along with 150,911 sex-, age-, and calendar year-matched controls. The severity level of non-fatal injuries was derived from the International Classification of Diseases Programs for Injury Categorization based on the diagnostic codes of National Health Insurance claims within 3 days after an MVC. Information on fatal injuries within 3 days after an MVC was obtained from the Taiwan Death Registry. Logistic regression models were used to estimate the odds ratios (ORs) and the corresponding 95% confidence intervals (CIs) of injury severity in association with type 2 diabetes.

**RESULTS:**

After adjusting for potential confounders, driver victims with type 2 diabetes experienced significantly higher risks of mild and severe non-fatal injuries than their counterparts without diabetes, with covariate-adjusted ORs of 1.08 (95% CI, 1.05 to 1.11) and 1.28 (95% CI, 1.20 to 1.37), respectively. By contrast, the adjusted OR for fatal injuries was not significantly elevated, at 1.02 (95% CI, 0.89 to 1.18). Similar results were found when car and scooter driver victims were analyzed separately.

**CONCLUSIONS:**

Type 2 diabetes was found to moderately increase the severity of non-fatal injuries from MVCs among car and scooter driver victims.

## INTRODUCTION

Patients with diabetes mellitus tend to have various health conditions that may impair their driving ability, including polyneuropathy, retinopathy, amputation, and vascular diseases [1,2]. Moreover, transient cognitive dysfunction or loss of consciousness from certain antihyperglycemic drug-induced sleep apnea and hypoglycemia further adversely affect driving performance [3]. Safe driving is dependent on many functions that can be impaired by hypoglycemia, with impaired driving performance and safety regulation violations being observed during hypoglycemic states in driving simulation studies [4]. Additionally, hypoglycemia can lead to loss of control, behavioral disorders, impaired consciousness, or even syncope, which may result in serious motor vehicle crashes (MVCs).

Although several population-based epidemiological studies have investigated driving impairment and collision risk in relation to diabetes, the results have often been contradictory [2,5]. This inconsistency is primarily due to heterogeneity in the design and samples adopted in these studies. For example, some drivers with diabetes may restrict or cease their driving activities due to deteriorating eyesight and cognition [6]. In addition, the road traffic crash rate is largely influenced by young people, particularly male drivers, a population in which the prevalence of diabetes is considerably lower than in older age groups [7]. Nonetheless, simulation studies have clearly demonstrated that people with diabetes tend to exhibit poor performance in muscle functioning, ankle proprioception, and accelerator pedal control [8]. Moreover, a study found that compared to individuals without diabetes who had equal confidence in their driving skills, patients with type 2 diabetes exhibited worse driving performance, as evidenced by a larger centerline deviation, a longer brake reaction time, and a shorter minimum time to collision [9].

While diabetes may increase the number of crashes (crash involvement) due to impaired driving performance, crashes involving individuals with diabetes might also be more serious (crash severity) because of poor judgment among drivers with diabetes. Moreover, patients with diabetes tend to have certain health conditions (e.g., hypoglycemia, depression, and impaired cognition) that could affect their consciousness as drivers, impair their control of the car, and possibly result in high-speed (i.e., high-energy) collisions. Most previous studies on this topic have investigated whether the risk of MVCs was higher in patients with diabetes, but very few population-based studies, if any, have examined whether and to what extent diabetes is associated with a greater severity of injury from MVCs [10]. The aim of this population-based cohort study was to examine whether type 2 diabetes is associated with a greater severity of injury among driver victims of MVCs.

## MATERIALS AND METHODS

### Source of data

Data from the Police-Reported Traffic Accident Registry (PTAR) (2008–2017), National Health Insurance (NHI) medical claims (2014–2017), and Taiwan Death Registry (TDR) (2015–2018) were analyzed in this study. The PTAR is managed by the National Police Agency of Taiwan. After a road traffic crash is made known to the police department, certified police accident investigators examine the accident scene and complete accident reports, which contain information relevant to the MVC [11,12]. NHI medical claims are retrieved from Taiwan’s NHI program; this program is implemented and supervised by the National Health Insurance Administration, which also performs quarterly expert reviews on random samples of medical claims to ensure their accuracy [13]. In Taiwan, all live births and deaths are legally required to be registered within 10 days after birth or death. The completeness of the TDR has been evaluated and considered high [14].

The data analyzed in this study, including the PTAR, NHI, and TDR databases, can be interlinked at the individual level using personal identifiers. Access to the aforementioned databases was approved by the Health and Welfare Data Science Center (HWDSC) of the Ministry of Health and Welfare. To protect the data, the data management and statistical analyses involved in this study were conducted on-site at the HWDSC.

### Design and participants

This retrospective cohort study initially included all 5,367,962 MVC events retrieved from the PTAR between 2008 and 2017. After excluding duplicate records and MVC events involving passengers or pedestrians, vehicle types other than cars or scooters, and driver victims aged <18 years, 4,564,639 MVC events by 3,599,576 adult driver victims remained. We further limited driver victims to those who had MVC events (n=1,137,577) in 2015–2017, and kept the first MVC event (i.e., index MVC event) for victims with multiple MVC events during this period. The utilization of data from more recent years may provide up-to-date information. By linking to the NHI inpatient/outpatient claims, we identified 75,737 driver victims with type 2 diabetes diagnosis codes (International Classification of Diseases, 9th revision, Clinical Modification [ICD-9-CM] 250.×0, ICD-9-CM 250.×2, or International Classification of Diseases, 10th revision, Clinical Modification [ICD-10-CM] E11) in ≥2 outpatient claims or ≥1 inpatient claims within a 1-year period prior to the index MVC. Limiting type 2 diabetes patients to those with ≥2 outpatient claims or ≥1 inpatient claims within a 1-year period avoided erroneous disease coding in medical claims [13]. Because the earliest inpatient/outpatient data of NHI available in this study were those claims made in 2005, we were able to determine the duration of type 2 diabetes based on the claim data.

**Figure 1:**
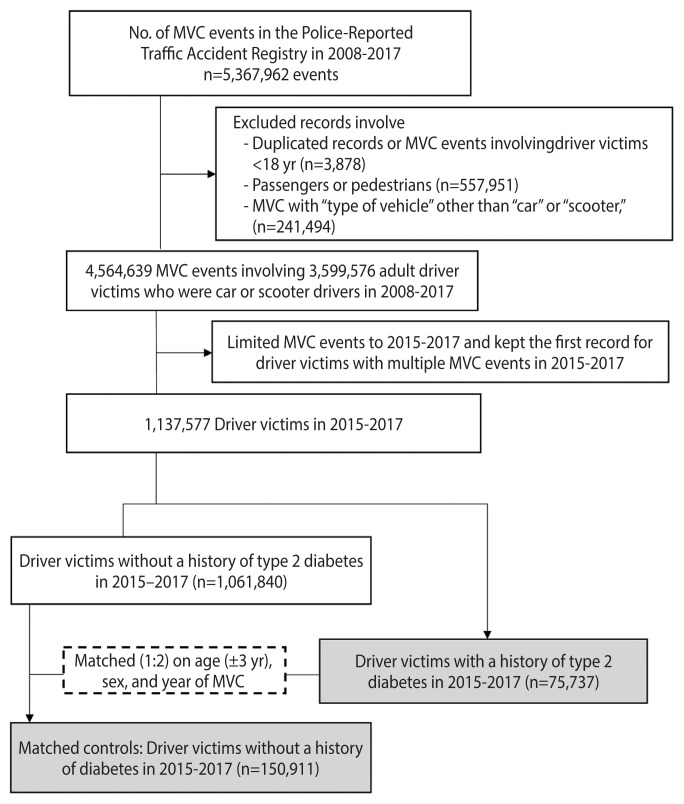
Flowchart of enrolling the study participants. MVC, motor vehicle crash.

For each type 2 diabetes driver victim, we randomly selected 2 control driver victims by matching age (±3 years), sex, and calendar year of the index MVC event. Eligible controls were required to have been alive on the day of the index MVC event and to have been free from any clinical diagnoses of type 1 or type 2 diabetes within a 3-year period prior to the date of the index MVC event. In total, 150,911 control driver victims were selected. A flowchart of study participant enrollment is shown in Figure 1.

### Outcome variables

The outcome variable of this study was categorized into fatal injuries, mild non-fatal injuries, severe non-fatal injuries, and no injuries (reference outcome). The study cohort was linked to the TDR for possible fatal injuries within 3 days after an MVC. Non-fatal injuries were determined according to the International Classification of Diseases Programs for Injury Categorization (ICDPIC), a statistical program based on the ICD-9-CM or ICD-10-CM diagnostic codes of Taiwan’s NHI claims [15]. For each injured individual, the ICDPIC determined the Maximum Abbreviated Injury Scale (MAIS) on the basis of the diagnostic codes from NHI emergency and inpatient claims within 3 days after an MVC. The MAIS classification considers the 3 most severe types of injuries and 7 body regions (i.e., head, neck, face, thorax, abdomen, extremity, and external). Individuals with an MAIS score of ≥3 were considered severely injured, and those with an MAIS score of 1 or 2 were classified as mildly injured. The reference outcome (i.e., no injury) included people who had no clinical visits or made clinical visits but showed an MAIS score of 0 within 3 days after an MVC.

### Covariates

In addition to the matching variables, a number of potential confounders were considered, including the number of MVC events within 3 years prior to the index MVC, the Charlson comorbidity index (CCI) [16] based on inpatient/outpatient claims in one year prior to the index MVC, the urbanization status of the area of residence (obtained from NHI information), the geographic area of residence, and the median family income of the area of residence, were analyzed at the city district and township level. The urbanization level for each of the 316 city districts and townships in Taiwan was based on a composite index of population density, education, the proportion of elderly people, the proportion of the agricultural workforce, and the density of physicians [17]. Information on the median family income for each of the 316 cities and townships in 2015 was obtained from the Government Open Data ( http://data.gov.tw/node/17983).

Previous studies have shown an evident urban-rural disparity in the health consequences of road traffic crashes, in which higher MVC-related mortality was observed in less urbanized areas [18]. This finding is possibly attributed to greater exposure to severe crashes [19], higher crash speeds [20], and less access to healthcare, particularly longer emergency medical service responses and transport times to higher-level trauma centers, in less urbanized areas [21,22].

### Statistical analysis

We first compared the characteristics of driver victims with and without type 2 diabetes by using either the 2 independent samples t-test or the chi-square test. The covariate-adjusted odds ratios (ORs) and the corresponding 95% confidence intervals (CIs) of various levels of severity (i.e., mild non-fatal injuries, severe non-fatal injuries, and fatal injuries) associated with MVCs were estimated from a conditional multiple logistic regression model that sequentially adjusted the covariates listed in Table 1. Model 1 adjusted for sex, age, calendar year of the MVC, and the type of vehicle. Model 2 additionally adjusted for the urbanization status of residence, median family income quartile, and geographic area. Model 3 further included the CCI and past MVC event number in the regression equation. The analyses were further stratified in accordance with vehicle type (i.e., car or scooter).

Inter-correlations among some of the selected covariates raise a concern about collinearity among those covariates simultaneously adjusted in the model. We assessed this potential statistical problem by examining the variance inflation factors (VIFs) of all covariates in the full model, and found that all VIFs were <2, suggesting no sign of collinearity (Supplementary Material 1). The statistical analyses were conducted with SAS version 9.4 (SAS Institute Inc., Cary, NC, USA), and the level of significance was set as α=0.05.

### Ethics statement

This study was approved by the Institutional Review Board of National Cheng Kung University Hospital (No. B-ER-109-088).

## RESULTS

Table 1 compares the characteristics of driver victims with type 2 diabetes and the matched controls. The study sample was dominated by males, with a mean age of 59 years in both groups. Patients with type 2 diabetes had a higher MVC event number within the past 3 years and greater CCI scores. People with type 2 diabetes tended to live in the eastern part of Taiwan and on remote islands, with lower urbanization levels and family incomes. A higher proportion of scooter driver victims was observed in patients with type 2 diabetes than in controls (62.65 vs. 58.16%). Moreover, 26.44% of the patients with type 2 diabetes had the disease for more than 10 years.

Compared with the control victims, driver victims with type 2 diabetes had a higher risk of mild non-fatal injuries (52.71 vs. 59.80%), severe non-fatal injuries (2.63 vs. 3.41%), and fatal injuries (0.52 vs. 0.56%), with significantly elevated crude ORs of 1.38 (95% CI, 1.36 to 1.41), 1.58 (95% CI, 1.50 to 1.66), and 1.32 (95% CI, 1.17 to 1.49), respectively. Similar results were found in both car and scooter driver victims when they were analyzed separately (Table 2).

Both models 1 and 2 (Table 3) showed that driver victims with type 2 diabetes had significantly higher adjusted odds ratios (aORs) of non-fatal and fatal injuries after MVCs than their counterparts without diabetes. With further adjustment for the CCI and past MVC event number (model 3), the aORs for non-fatal and fatal injuries were all attenuated, but those for mild and severe non-fatal injuries were still significantly elevated, at 1.08 (95% CI, 1.05 to 1.11) and 1.28 (95% CI, 1.20 to 1.37), respectively. In contrast, the aOR for fatal injuries in association with type 2 diabetes was comparable to null statistically, at 1.02 (95% CI, 0.89 to 1.18). No clear evidence was found for dose-gradient relationship between type 2 diabetes disease duration and the aORs of non-fatal or fatal injuries (Table 3). The complete results from the multivariate logistic regression models are provided in Supplementary Material 1.

Table 3 also presents vehicle type-specific analyses. Among car driver victims with type 2 diabetes, the aOR for mild non-fatal injuries was significantly higher than among their counterparts without diabetes, at 1.08 (95% CI, 1.04 to 1.13). The aORs for severe non-fatal injuries (1.17; 95% CI, 0.88 to 1.55) and fatal injuries (1.26; 95% CI, 0.84 to 1.89), in contrast, were not significantly elevated. The corresponding aORs of mild non-fatal injuries, severe non-fatal injuries, and fatal injuries were 1.06 (95% CI, 1.02 to 1.10), 1.27 (95% CI, 1.18 to 1.36), and 0.98 (95% CI, 0.84 to 1.14).

## DISCUSSION

This study found increased risks of mild and severe non-fatal injuries among driver victims with type 2 diabetes mellitus that were independent of socio-demographic characteristics, comorbidities, and the urbanization level or geographic area of residence. However, type 2 diabetes did not significantly increase the risk of fatal injuries within 3 days after a vehicle collision. Additionally, similar risk estimations were observed for car and scooter victims with type 2 diabetes. This study provides further insights into the outcomes of MVCs among driver victims with type 2 diabetes.

Patients with type 2 diabetes frequently have a limited awareness of hypoglycemia and a lack of knowledge that certain diabetes-related health conditions, such as depression and impaired cognition, may increase the risk of MVCs. Hypoglycemia is a risk factor for driving-related traumatic injuries in patients with diabetes; it poses a significant burden as a risk factor and predictor of poor outcomes of traumatic injuries [23]. Previous studies showed increased risks of depression and impaired cognition in patients with type 2 diabetes [24,25], and both health conditions were associated with a higher risk of MVCs. In a driving simulation study, Bulmash et al. [26] reported that depression impaired driving behavior, as shown by slower steering reaction times and a greater number of crashes. In a meta-analysis of 113 studies, major depressive disorder was found to be associated with impaired performance on neuropsychological measures of executive function [27]. In addition, Alzheimer’s disease patients were found to make more turning mistakes and lose orientation while driving even in the early stage of disease [28]. Moreover, a meta-analysis showed that about 14% of patients with very mild dementia and 33% with mild dementia failed an on-road driving test, whereas only 1.6% failed in the control group [29].

The above-mentioned health conditions may affect drivers’ consciousness and impair their control of cars, possibly resulting in high-speed (i.e., high-energy) collisions. Additionally, Ma et al. [9] conducted a simulation study and found that compared with healthy individuals, drivers with type 2 diabetes exhibited worse driving performance, as evidenced by larger centerline deviations, longer brake reaction times, and shorter minimum times to collision. Collisions with higher energy may be partly responsible for the increased risks of mild and severe non-fatal injuries among driver victims with type 2 diabetes noted in the current study.

In addition to the energy of collisions, patients with diabetes may also face a higher risk of sustaining fractures after MVCs. A recent meta-analysis of 37 studies with 3,123,382 participants revealed a pooled relative risk of 1.5 (95% CI, 1.3 to 1.8) for any fracture in patients with diabetes [30]. This meta-analysis also showed that diabetes is an independent risk factor for low-energy fractures, and this relationship was more pronounced for hip fractures [30]. In an earlier meta-analysis, Wang et al. [31] reported that patients with diabetes experienced higher risks of total, hip, upper arm, and ankle fractures, with type 1 diabetes having a more harmful effect than type 2 diabetes. Vulnerability to fractures among patients with diabetes could have also contributed to the increased risk of severe injuries among driver victims with type 2 diabetes.

Patients with a longer duration of diabetes are expected to suffer more diabetic complications [14] and other comorbidities (e.g., hypoglycemia and fractures), possibly increasing injury severity after MVCs. However, our results did not demonstrate a dose-gradient relationship between type 2 diabetes duration and injury severity. Our study did show that the crude ORs of mild non-fatal injuries and fatal injuries increased with the duration of diabetes, but this dose-relationship disappeared after adjusting for the CCI and past MVC event number, which highlights the importance of comorbidities in increasing the risk of injury after MVCs. Additionally, advances in diabetes care technology and a better understanding of the safety consequences of diabetes have expanded available techniques to limit the risks of driving with diabetes and the consequences of collisions, likely diluting the adverse effect of longer type 2 diabetes duration on injury severity from MVCs [32].

Our study did not show a significant increase in the risk of fatal injuries within 3 days after MVCs among driver victims with type 2 diabetes. Diabetes significantly increases rates of malunion, infection, and reoperation in patients with surgically treated lower extremity fractures, which is a common condition that results from MVCs [33]. Diabetes also substantially alters bone metabolism and soft tissue healing, posing a risk of adverse fracture healing and other complications [33]. A recent meta-analysis reported that the overall complication risk after an ankle fracture was nearly 2 times higher among patients with diabetes than among individuals without diabetes (OR, 1.9; 95% CI, 1.7 to 2.0). The complication risk was even higher in patients with advanced diabetes (OR, 8.4; 95% CI, 2.9 to 24.5). Moreover, the risk of infection was over 3 times higher in patients with diabetes than in individuals without diabetes (OR, 3.4; 95% CI, 2.9 to 9.8) [34]. Alkhouli & Alqahtani [35] reported considerably higher in-hospital mortality among patients who were in MVCs and had experienced acute myocardial infarction (AMI), a cardiovascular complication of diabetes, than MVC victims without AMI (21.7 vs. 2.7%). Although strong associations were previously noted between MVCs and certain medical conditions, our study showed only a mild increase in the risk of non-fatal injuries probably because we used the Abbreviated Injury Scale (AIS) severity score, which is an ordinal scale of 1 to 6 (1 indicating a minor injury and 6 being maximal). A person who sustains an injury with a score of 3 or higher on the AIS is classified as clinically seriously injured (MAIS 3+) [15]. Our data showed only 2.8% of all participants in this study were categorized as MAIS 3+. A small variation in the AIS severity score resulted in smaller relative risk estimates.

In fact, our data showed a significantly higher risk of fatal injuries within 3 days after an MVC, with a crude OR of 1.32 (95% CI, 1.17 to 1.49). In the sequential adjustment for covariates, we found that the significantly increased risk of fatal injuries persisted after adjustment for socio-demographic characteristics, vehicle type, and urbanization. Nonetheless, the significant increase in 3-day fatal injuries disappeared after further adjustment for the CCI and past MVC event number. The CCI could serve as proxy measures of the aforementioned medical conditions that contributed to the increased risk of fatal injuries after MVCs.

The strengths of the current study include the large number of study participants, which allowed adequate statistical power for detecting a small magnitude of increased risks of mild and severe non-fatal injuries. The large sample size also permitted the assessment of dose-response relationships between type 2 diabetes duration and injury severity, along with separate analyses of car and scooter driver victims. Second, the study cohort (i.e., driver victims) was retrieved from the PTAR, which is a population-based registry that covers nearly all road traffic crashes, including those without injuries. An analysis limited to a comparison between driver victims with and without diabetes in hospital settings is likely to be subject to referral bias because driver victims who require treatment after MVCs tend to exhibit smaller variability in injury severity [12]. Nonetheless, the police-reported road traffic crash data could have under-reported some minor motor vehicle collisions that involved no injury or property damage or loss [36]. Third, this study interlinked data among the PTAR, NHI medical claims, and the TDR databases, considerably reducing the likelihood of incomplete information due to follow-up loss.

Despite the aforementioned strengths, several weaknesses of this study should be noted. First, sole reliance on diagnostic codes to identify patients with type 2 diabetes may be subject to disease misclassification because a proportion of people with type 2 diabetes may remain underdiagnosed. However, we limited type 2 diabetes to those with ≥2 outpatient claims or ≥1 inpatient claim within 1 year prior to the MVC, largely avoiding erroneous type 2 diabetes coding in medical claims and disease misclassification bias [13]. Moreover, disease misclassification (if any) in this study is likely to be non-differential, leading to an underestimation rather than an overestimation of the true association of type 2 diabetes with injury severity after MVCs. Second, due to a lack of detailed information on the potential mechanisms involving MVCs, such as the use of a seatbelt or other restraint equipment, vehicle speed at the time of the crash, and the time of transportation from the crash scene to the medical institution, we were unable to consider these variables in the analyses, which of course limits the specific interpretations of the study findings. Third, we ascertained MVC-related fatal and non-fatal injuries in a 3-day period after collisions to assure that the injuries captured were likely to have been caused by the corresponding MVC. A study investigated the time of death from vehicle crashes in the 1990s and showed that 46% of deaths occurred within 30 minutes, 24% between 30 minutes and 1.5 hours, and 90% within 24 hours [37]. In addition, a recent Australian study defined vehicle crash-related hospital admissions as those happening on the same day or within 1 day of a record in the police-reported crash data [38]. Nonetheless, the comparability of our study findings with international data is limited because most major road safety-related organizations define traffic accident deaths as deaths within 30 days after the accident [39]. Fourth, this study confined the analysis to driver victims with type 2 diabetes, which is likely to have underestimated the overall burden of type 2 diabetes-related MVCs because people with type 2 diabetes could have been present among other traffic crash victims, such as pedestrians and passengers. Additionally, the opponent vehicle drivers who might not have type 2 diabetes could also be victims of the MVCs. Fifth, the generalizability of the study results from Taiwan to other countries may not be straightforward because of possible dissimilarities in road conditions, emergency medical service provision, and health care systems among countries.

We noted a small increase in the risks of mild and severe non-fatal injuries among driver victims with type 2 diabetes after MVCs. In addition, our data showed that patients with type 2 diabetes in Taiwan tended to have a lower socioeconomic background, more rural residence areas, and a higher proportion of scooter accidents. These socioeconomic and urban-rural inequalities in MVCs associated with type 2 diabetes may be a concern from both research and policy points of view.

## Figures and Tables

**Table 1:** Comparison of characteristics between driver victims with and without type 2 diabetes

Characteristics	Type 2 diabetes	Matched controls	p-value
Total	75,464 (100)	150,911 (100)	

Sex^1^			
Male	51,699 (68.51)	103,398 (68.52)	0.970
Female	23,765 (31.49)	47,513 (31.48)	

Age (yr)^1^			
<45	8,808 (11.67)	17,946 (11.89)	0.175
45–54	15,082 (19.99)	29,947 (19.84)	
55–64	25,923 (34.35)	51,355 (34.03)	
≥65	25,651 (33.99)	51,663 (34.23)	
Mean±SD	59.07±12.13	59.18±12.39	0.044

Calendar year of MVCs^1^			
2015	25,589 (33.91)	51,176 (33.91)	>0.999
2016	25,416 (33.68)	50,828 (33.68)	
2017	24,459 (32.41)	48,907 (32.41)	

Past MVC event (n)^2^			
0	74,722 (99.02)	149,595 (99.13)	0.005
1	698 (0.92)	1,262 (0.84)	
≥2	44 (0.06)	54 (0.04)	

CCI^3^			<0.001
0	3,833 (5.08)	84,166 (55.77)	
1	40,091 (53.13)	52,586 (34.85)	
≥2	31,540 (41.79)	14,159 (9.38)	

Urbanization status of residence area			
Urban	22,451 (29.75)	47,550 (31.51)	<0.001
Satellite	24,902 (33.00)	49,933 (33.09)	
Rural	28,111 (37.25)	53,428 (35.41)	

Median family income quartile^4^			
Min-Q1	19,727 (26.14)	37,377 (24.77)	<0.001
Q1–Q3	36,809 (48.78)	72,050 (47.74)	
Q3-Max	18,928 (25.08)	41,484 (27.49)	

Geographic area of residence			
North	25,176 (33.36)	52,855 (35.02)	<0.001
Central	21,707 (28.76)	43,771 (29.00)	
South	25,510 (33.80)	48,765 (32.31)	
East and islands	3,071 (4.07)	5,520 (3.66)	

Type of vehicle			
Car	28,185 (37.35)	63,147 (41.84)	<0.001
Scooter	47,279 (62.65)	87,764 (58.16)	

Type 2 diabetes duration (yr)			
None	0 (0.00)	150,911 (100)	<0.001
0–4	24,190 (32.06)	0 (0.00)	
5–9	31,323 (41.51)	0 (0.00)	
≥10	19,951 (26.44)	0 (0.00)	

Values are presented as number (%).

MVC, motor vehicle crash; CCI, Charlson comorbidity index; Min, minimum; Max, maximum; NTD, New Taiwan dollars.

1 Matching variables.

2 Within a 3-year period prior to the index (first) MVC in 2015–2017.

3 Based on inpatient/outpatient claims in a 1-year period prior to the index (first) MVC in 2015–2017.

4 Q1=565,000 NTD, Q3=642,000 NTD; 1 USD 28 NTD.

**Table 2:** Vehicle type-specific number of injuries and crude odds ratios of injury severity in relation to type 2 diabetes among driver victims of MVCs

Variables	Total no. of study subjects	No. of subjects injured by level of severity						

		Reference^1^	Non-fatal injuries^2^		Fatal injuries^3^	Non-fatal injuries^2^		Fatal injuries^3^
	
			Mild	Severe		Mild	Severe	
Car and scooter driver victims								
Total	226,375	93,951	124,677	6,548	1,199	-	-	-
Type 2 diabetes								
No	150,911	66,607	79,552	3,974	778	1.00 (reference)	1.00 (reference)	1.00 (reference)
Yes	75,464	27,344	45,125	2,574	421	1.38 (1.36, 1.41)	1.58 (1.50, 1.66)	1.32 (1.17, 1.49)
Duration (yr)								
0–4	24,190	9,565	13,757	760	108	1.20 (1.17, 1.24)	1.33 (1.23, 1.44)	0.97 (0.79, 1.18)
5–9	31,323	11,333	18,401	1,418	171	1.36 (1.33, 1.39)	2.10 (1.97, 2.24)	1.29 (1.09, 1.53)
≥10	19,951	6,446	12,967	396	142	1.68 (1.63, 1.74)	1.03 (0.93, 1.15)	1.89 (1.57, 2.26)

Car driver victims								
Total	91,332	70,264	20,597	323	148	-	-	-
Type 2 diabetes								
No	63,147	49,962	12,888	207	90	1.00 (reference)	1.00 (reference)	1.00 (reference)
Yes	28,185	20,302	7,709	116	58	1.47 (1.42, 1.52)	1.38 (1.10, 1.73)	1.59 (1.14, 2.21)
Duration (yr)								
0–4	9,760	7,208	2,483	43	26	1.34 (1.27, 1.40)	1.44 (1.04, 2.00)	2.00 (1.29, 3.10)
5–9	11,672	8,433	3,168	52	19	1.46 (1.39, 1.52)	1.49 (1.10, 2.02)	1.25 (0.76, 2.05)
≥10	6,753	4,661	2,058	21	13	1.71 (1.62, 1.81)	1.09 (0.69, 1.71)	1.55 (0.86, 2.77)

Scooter driver victims								
Total	135,043	23,687	104,080	6,225	1,051	-	-	-
Type 2 diabetes								
No	87,764	16,645	66,664	3,767	688	1.00 (reference)	1.00 (reference)	1.00 (reference)
Yes	47,279	7,042	37,416	2,458	363	1.33 (1.29, 1.37)	1.54 (1.46, 1.63)	1.25 (1.09, 1.42)
Duration (yr)								
0–4	14,430	2,357	11,274	717	82	1.19 (1.14, 1.25)	1.34 (1.23, 1.47)	0.84 (0.67, 1.06)
5–9	19,651	2,900	15,233	1,366	152	1.31 (1.26, 1.37)	2.08 (1.93, 2.24)	1.27 (1.06, 1.52)
≥10	13,198	1,785	10,909	375	129	1.53 (1.45, 1.61)	0.93 (0.83, 1.04)	1.75 (1.44, 2.12)

Values are presented as odds ratio (95% confidence interval).

MVC, motor vehicle crash; MAIS, Maximum Abbreviated Injury Scale.

1 The reference outcome included driver victims who had no clinical visits or who made clinical visits but showed a MAIS score of 0 (i.e., no injury diagnostic codes) in 3 days after a MVC; Driver victims with the reference outcome were also not dead in 3 days after the crash.

2 Classification based on the MAIS derived from medical claims.

3 Death occurring within 3 days after a MVC.

**Table 3:** Covariate-adjusted odds ratios and 95% confidence intervals of injury severity in relation to type 2 diabetes among driver victims of MVCs

Variables	Model 1^1^			Model 2^2^			Model 3^3^		
		
	Non-fatal injuries^4^		Fatal injuries^5^	Non-fatal injuries^4^		Fatal injuries^5^	Non-fatal injuries^4^		Fatal injuries^5^
		
	Mild	Severe		Mild	Severe		Mild	Severe	
Car and scooter driver victims									
Type 2 diabetes									
No	1.00 (reference)	1.00 (reference)	1.00 (reference)	1.00 (reference)	1.00 (reference)	1.00 (reference)	1.00 (reference)	1.00 (reference)	1.00 (reference)
Yes	1.30 (1.27, 1.33)	1.50 (1.42, 1.59)	1.27 (1.12, 1.44)	1.42 (1.38, 1.45)	1.63 (1.54, 1.73)	1.35 (1.20, 1.53)	1.08 (1.05, 1.11)	1.28 (1.20. 1.37)	1.02 (0.89, 1.18)
Duration (yr)									
0–4	1.26 (1.22,1.31)	1.46 (1.33, 1.59)	1.18 (0.96, 1.45)	1.25 (1.21, 1.30)	1.44 (1.31, 1.57)	1.14 (0.93, 1.41)	1.10 (1.06, 1.14)	1.29 (1.18, 1.42)	0.99 (0.80, 1.23)
5–9	1.27 (1.23, 1.32)	1.53 (1.42, 1.64)	1.18 (1.00, 1.41)	1.27 (1.23, 1.31)	1.51 (1.41, 1.63)	1.16 (0.98, 1.39)	1.04 (1.00, 1.08)	1.29 (1.19, 1.40)	0.94 (0.78, 1.14)
≥10	1.40 (1.34, 1.46)	1.42 (1.26, 1.60)	1.50 (1.24, 1.81)	1.39 (1.34, 1.45)	1.42 (1.26, 1.59)	1.50 (1.24, 1.81)	1.11 (1.06, 1.16)	1.17 (1.03, 1.32)	1.17 (0.96, 1.44)

Car driver victims									
Total									
Type 2 diabetes									
No	1.00 (reference)	1.00 (reference)	1.00 (reference)	1.00 (reference)	1.00 (reference)	1.00 (reference)	1.00 (reference)	1.00 (reference)	1.00 (reference)
Yes	1.39 (1.34, 1.43)	1.32 (1.05, 1.66)	1.48 (1.06, 2.08)	1.48 (1.43, 1.53)	1.40 (1.11, 1.76)	1.54 (1.10, 2.16)	1.08 (1.04, 1.13)	1.17 (0.88, 1.55)	1.26 (0.84, 1.89)
Duration (yr)									
0–4	1.32 (1.25, 1.39)	1.47 (1.05, 2.05)	2.16 (1.38, 3.39)	1.41 (1.34, 1.49)	1.54 (1.11, 2.16)	2.23 (1.42, 3.49)	1.08 (1.03, 1.14)	1.34 (0.93, 1.92)	1.87 (1.15, 3.03)
5–9	1.37 (1.30, 1.43)	1.18 (0.87, 1.61)	1.12 (0.67, 1.88)	1.46 (1.40, 1.53)	1.26 (0.93, 1.72)	1.19 (0.71, 1.98)	1.05 (1.00, 1.11)	1.04 (0.73, 1.47)	0.94 (0.54, 1.64)
≥10	1.51 (1.43, 1.60)	1.40 (0.87, 2.24)	1.28 (0.71, 2.31)	1.61 (1.52, 1.71)	1.49 (0.93, 2.39)	1.32 (0.73, 2.38)	1.13 (1.06, 1.20)	1.20 (0.72, 1.99)	1.01 (0.53, 1.93)

Scooter driver victims									
Total									
Type 2 diabetes									
No	1.00 (reference)	1.00 (reference)	1.00 (reference)	1.00 (reference)	1.00 (reference)	1.00 (reference)	1.00 (reference)	1.00 (reference)	1.00 (reference)
Yes	1.21 (1.17, 1.25)	1.42 (1.34, 1.51)	1.17 (1.03, 1.34)	1.35 (1.30, 1.39)	1.58 (1.48, 1.68)	1.27 (1.11, 1.45)	1.06 (1.02, 1.10)	1.27 (1.18, 1.36)	0.98 (0.84, 1.14)
Duration (yr)									
0–4	1.20 (1.14, 1.26)	1.39 (1.26, 1.53)	0.99 (0.78, 1.25)	1.34 (1.27, 1.41)	1.55 (1.40, 1.70)	1.06 (0.84, 1.35)	1.10 (1.04, 1.16)	1.29 (1.16, 1.43)	0.86 (0.67, 1.10)
5–9	1.18 (1.13, 1.24)	1.45 (1.35, 1.57)	1.12 (0.93, 1.35)	1.31 (1.25, 1.37)	1.61 (1.49, 1.74)	1.21 (1.01, 1.46)	1.01 (0.96, 1.07)	1.28 (1.17, 1.39)	0.93 (0.76, 1.13)
≥10	1.28 (1.21, 1.36)	1.32 (1.17, 1.50)	1.42 (1.16, 1.74)	1.41 (1.33, 1.49)	1.46 (1.29, 1.65)	1.56 (1.27, 1.9)	1.07 (1.01, 1.14)	1.13 (0.99, 1.29)	1.16 (0.93, 1.44)

MVC, motor vehicle crash; MAIS, Maximum Abbreviated Injury Scale.

1 Model 1 adjusted for sex, age, calendar year of the MVC, and type of vehicle.

2 In addition to the covariates adjusted in model 1, model 2 additionally adjusted for urbanization status of residence, median family income quartile, and geographic area.

3 In addition to the covariates adjusted in model 2, model 3 additionally adjusted for the Charlson comorbidity index and past MVC event number.

4 Classification based on the MAIS derived from medical claims.

5 Fatal injury that occurred in 3 days after an MVC.

## SUPPLEMENTARY MATERIALS

Supplementary Material 1. Covariate aORs of injury severity and death in relation to type 2 diabetes and selected covariates among driver victims of MVCs
