# Supplementary material for: Type 2 diabetes mellitus increases the severity of non-fatal injuries, but not the risk of fatal injuries, among driver victims of motor vehicle crashes in Taiwan
Source: Epidemiol Health. 2022 Sep 16;44:e2022076. doi: 10.4178/epih.e2022076 (PMC10089709; doi:10.4178/epih.e2022076)
Supplement: Supplementary Material 1. — Covariate aORs of injury severity and death in relation to type 2 diabetes and selected covariates among driver victims of MVCs [file epih-44-e2022076-Supplementary-1.docx]

**Supplementary Material 1.** Covariate aORs of injury severity and death in relation to type 2 diabetes and selected covariates among driver victims of MVCs

|  | Total number of study subjects | Number of subjects injured by level of severity | | | |  |  | aOR (95% CI)^1^ |  | VIF^1^ |
| --- | --- | --- | --- | --- | --- | --- | --- | --- | --- | --- |
|  |  | Reference^2^ | Non-fatal injury | | Fatal injury |  | Non-fatal injury | | Fatal injury |  |
|  |  |  | Mild | Severe |  |  | Mild | Severe |  |  |
|  | *N* | *n* | *n* | *n* | *n* |  |  |  |  |  |
| Total ^3^ | 226,375 | 93,951 | 124,677 | 6,548 | 1,199 |  |  |  |  |  |
| Type 2 diabetes |  |  |  |  |  |  |  |  |  |  |
| No | 150,911 | 66,607 | 79,552 | 3,974 | 778 |  | 1.00 | 1.00 | 1.00 | 1.39 |
| Yes | 75,464 | 27,344 | 45,125 | 2,574 | 421 |  | 1.08(1.05-1.11) | 1.28(1.20-1.37) | 1.02(0.89-1.18) |  |
| Duration (yrs) |  |  |  |  |  |  |  |  |  |  |
| 0–4 | 24,190 | 9,565 | 13,757 | 760 | 108 |  | 1.10(1.06-1.14) | 1.29(1.18-1.42) | 0.99(0.80-1.23) |  |
| 5–9 | 31,323 | 11,333 | 18,401 | 1,418 | 171 |  | 1.04(1.00-1.08) | 1.29(1.19-1.40) | 0.94(0.78-1.14) |  |
| 10+ | 19,951 | 6,446 | 12,967 | 396 | 142 |  | 1.11(1.06-1.16) | 1.17(1.03-1.32) | 1.17(0.96-1.44) |  |
| Gender |  |  |  |  |  |  |  |  |  | 1.08 |
| Male | 155,097 | 76,605 | 73,807 | 3,808 | 877 |  | 1.00 | 1.00 | 1.00 |  |
| Female | 71,278 | 17,346 | 50,870 | 2,740 | 322 |  | 1.86(1.81–1.91) | 1.79(1.69–1.90) | 0.99(0.87–1.14) |  |
| Age (years) |  |  |  |  |  |  |  |  |  | 1.08 |
| <45 | 26,754 | 13,310 | 12,916 | 470 | 58 |  | 1.00 | 1.00 | 1.00 |  |
| 45–54 | 45,029 | 24,159 | 19,894 | 854 | 122 |  | 1.00(0.96–1.04) | 1.22(1.08–1.38) | 1.32(0.96–1.83) |  |
| 55–64 | 77,278 | 34,468 | 40,474 | 2,037 | 299 |  | 1.18(1.14–1.23) | 1.68(1.51–1.88) | 1.90(1.42–2.53) |  |
| ≥65 | 77,314 | 22,014 | 51,393 | 3,187 | 720 |  | 1.71(1.65–1.78) | 2.99(2.68–3.34) | 4.47(3.38–5.92) |  |
| Calendar year of MVCs |  |  |  |  |  |  |  |  |  | 1.04 |
| 2015 | 76,765 | 31,657 | 39,985 | 4,711 | 412 |  | 1.00 | 1.00 | 1.00 |  |
| 2016 | 76,244 | 31,869 | 43,006 | 963 | 406 |  | 1.05(1.02–1.08) | 0.19(0.18–0.21) | 0.94(0.81–1.08) |  |
| 2017 | 73,366 | 30,425 | 41,686 | 874 | 381 |  | 1.05(1.02–1.08) | 0.18(0.17–0.20) | 0.95(0.81–1.10) |  |
| Past MVC event number ^4^ |  |  |  |  |  |  |  |  |  | 1.00 |
| 0 | 224,317 | 93,139 | 123,501 | 6,491 | 1,186 |  | 1.00 | 1.00 | 1.00 |  |
| 1 | 1,960 | 812 | 1176 | 57 | 13 |  | 1.05(0.93–1.18) | 1.01(0.76–1.36) | 1.20(0.67–2.15) |  |
| 2+ | 98 | ^7^ | ^7^ | ^7^ | ^7^ |  | 0.65(0.39–1.08) | 0.29(0.04–2.16) | ^8^ |  |
| CCI ^5^ |  |  |  |  |  |  |  |  |  | 1.47 |
| 0 | 87,999 | 43,535 | 42,201 | 1,900 | 363 |  | 1.30(1.26–1.34) | 1.23(1.14–1.32) | 1.33(1.14–1.55) |  |
| 1 | 92,677 | 36,351 | 52,987 | 2,833 | 506 |  | 1.70(1.64–1.77) | 1.56(1.43–1.71) | 1.76(1.47–2.12) |  |
| >=2 | 45,699 | 14,065 | 29,489 | 1,815 | 330 |  | 1.30(1.26–1.34) | 1.23(1.14–1.32) | 1.33(1.14–1.55) |  |
| Urbanization status of residence |  |  |  |  |  |  |  |  |  | 1.48 |
| Urban | 70,001 | 31,632 | 36,454 | 1,729 | 186 |  | 1.00 | 1.00 | 1.00 |  |
| Satellite | 74,835 | 32,397 | 40,181 | 1,932 | 325 |  | 1.04(1.01–1.07) | 0.99(0.91–1.08) | 1.37(1.11–1.69) |  |
| Rural | 77,329 | 28,372 | 45,542 | 2,746 | 669 |  | 1.37(1.32–1.42) | 1.68(1.54–1.83) | 2.86(2.33–3.52) |  |
| Median family income quartiles ^6^ |  |  |  |  |  |  |  |  |  | 1.51 |
| Min.–Q1 | 57,104 | 21,113 | 33,324 | 2,157 | 510 |  | 1.13(1.09–1.18) | 1.44(1.30–1.60) | 2.43(1.90–3.11) |  |
| Q1–Q3 | 108,859 | 44,323 | 60,908 | 3,087 | 541 |  | 1.01(0.98–1.04) | 1.13(1.03–1.23) | 1.51(1.21–1.88) |  |
| Q3–max. | 54,235 | 25,848 | 27,168 | 1,094 | 125 |  | 1.00 | 1.00 | 1.00 |  |
| Geographic area |  |  |  |  |  |  |  |  |  | 1.08 |
| North | 78,031 | 36,813 | 38,963 | 1,982 | 273 |  | 1.00 | 1.00 | 1.00 |  |
| Central | 65,478 | 25,675 | 37,406 | 2,021 | 376 |  | 1.06(1.02–1.09) | 0.87(0.80–0.94) | 0.85(0.70–1.02) |  |
| South | 74,275 | 27,981 | 43,573 | 2,243 | 478 |  | 1.06(1.03–1.10) | 0.88(0.81–0.94) | 1.24(1.05–1.46) |  |
| East and islands | 8,557 | 3,465 | 4,719 | 302 | 71 |  | 0.99(0.93–1.05) | 0.98(0.84–1.13) | 1.12(0.84–1.50) |  |
| Type of vehicle |  |  |  |  |  |  |  |  |  | 1.11 |
| Car | 91,332 | 70,264 | 20,597 | 323 | 148 |  | 1.00 | 1.00 | 1.00 |  |
| Scooter | 135,043 | 23,687 | 104,080 | 6,225 | 1,051 |  | 13.26(12.96–13.57) | 49.86(44.4–55.99) | 19.86(16.58–23.78) |  |

^1^ OR, odds ratio; CI, confidence interval; VIF, variance inflation factor

^2^. The reference outcome included driver victims who had no clinical visits or who made clinical visits but showed a maximum abbreviated injury scale (MAIS) score of 0 (i.e., no injury diagnostic codes) in 3 days after motor vehicle crashes. Driver victims with reference outcome were also not dead in 3 days after crashes.

^3^ Inconsistency between total number of study subjects and the number summed for some variables (urbanization status of residence, median family income quartiles, and geographic area of residence) was due to missing information.

^4^ Within a 3-year period prior to the index (first) MVC in 2015–2017

^5^ CCI, Charlson Comorbidity Index based on the inpatient / outpatient claims in a 1-year period prior to the index (first) MVC in 2015–2017

^6^ Q1=565,000 New Taiwan Dollars (NTD), Q3=642,000 NTD; 1 USD ≅ 28 NTD

^7^ The exact or calculated number below 3 is not specified, in accordance with Taiwanese privacy regulations.

^8^ Not calculable due to very limited number of event.
